# Tuberculosis of the Parietal Bone in an Indian Boy: A Rare Entity

**DOI:** 10.7759/cureus.55751

**Published:** 2024-03-07

**Authors:** Sankalp Yadav, Gautam Rawal, Madhan Jeyaraman, Naveen Jeyaraman

**Affiliations:** 1 Medicine, Shri Madan Lal Khurana Chest Clinic, New Delhi, IND; 2 Respiratory Medical Critical Care, Max Super Speciality Hospital, New Delhi, IND; 3 Clinical Research, Virginia Tech India, Dr MGR Educational and Research Institute, Chennai, IND; 4 Orthopaedics, ACS Medical College and Hospital, Dr MGR Educational and Research Institute, Chennai, IND

**Keywords:** calvarium defects, mycobacterium tuberculosis (mtb), tuberculosis, rarefaction, parietal bone

## Abstract

Tuberculosis is a common issue in endemic regions. The disease can affect both adults and children. Tuberculosis involving the flat bones of the skull is infrequently reported. Besides, reports of parietal bone tuberculosis in children are rare and a diagnostic challenge. Often, these cases report late, and this could compromise the treatment outcomes. Herein, a case of post-traumatic tuberculosis of the left parietal bone is presented in a child. The diagnosis was achieved by radiometric investigations and the isolation of *Mycobacterium tuberculosis *from the pus. He was initiated on antituberculous chemotherapy.

## Introduction

*Mycobacterium tuberculosis *bacteria cause tuberculosis, a chronic infectious disease that can affect nearly any part of the body, particularly the lungs [1]. Malnutrition, poor socioeconomic status, and immunodeficiency syndromes continue to make tuberculosis one of the most significant health challenges in developing countries [2].

While tuberculosis of the bones is relatively common, tuberculosis of the skull is less frequent compared to other skeletal locations [1]. Skeletal tuberculosis, comprising only 1-2% of cases, is a rare manifestation. Even rarer is primary calvarial tuberculosis, which constitutes only 0.2 to 1.3% of skeletal tuberculosis cases in patients where there is no evidence of tuberculosis elsewhere in the body [2]. There's a male predominance, with a male-to-female ratio of 2:1, and the majority of cases (75-80%) occur in individuals under 20 years old [3].

Herein, a case of a 14-year-old boy is presented who came with complaints of painful swelling on the left side of the skull with a discharging sinus. He was put on antituberculous treatment after a detailed clinical and diagnostic evaluation.

## Case presentation

A 14-year-old Indian boy reported with his father to the outpatient department with complaints of painful swelling with a discharging sinus for one month. He was all right 30 days ago when he hit the tap in his washroom. Initially, there was a painful swelling about 2 x 2 cm in size, but over the course of time, it grew to a significant size and was associated with discharging sinus. The discharge was yellow-colored, purulent, non-foul-smelling, and non-blood-tinged.

There was no history of tuberculosis in the family or any contacts. Also, there was no history of seizures, vomiting, or any constitutional signs of tuberculosis. Moreover, there was no history of any stays at slums, juvenile homes, or refugee camps.

A general examination was suggestive of a hemodynamically stable boy. A local examination revealed a swollen left parietal part of the skull; the swelling was 4 × 3 × 3 cm in size, soft, non-pulsatile, and fluctuant with ill-defined and diffuse borders. A small defect was palpable in the left parietal bone posterior to the swelling, with a discharging sinus. The surrounding skin was erythematous, but there were no engorged veins. Further, there was no pallor, clubbing, cyanosis, icterus, edema, or lymphadenopathy. His systemic examination was not suggestive of any disease.

**Figure 1:**
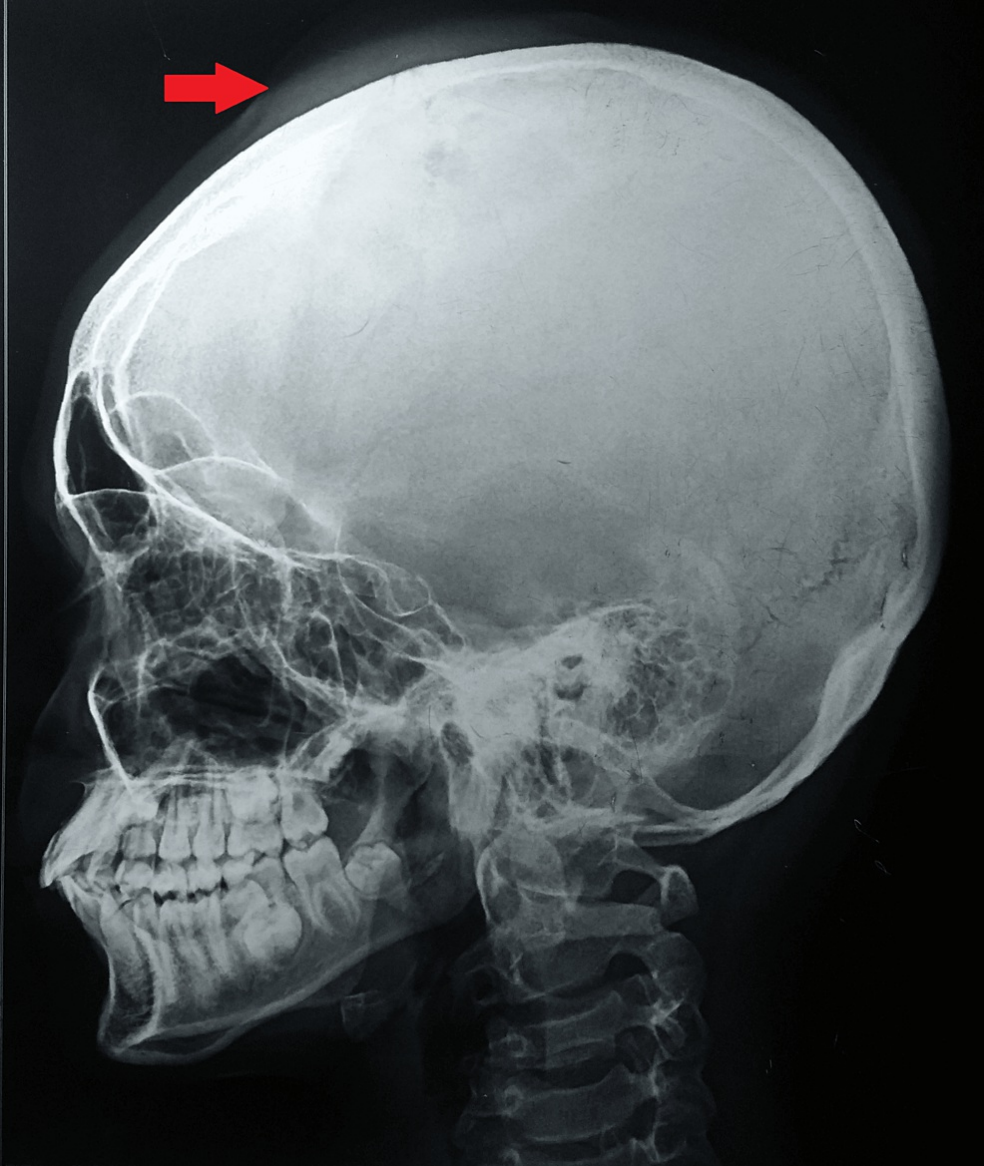
A plain radiograph of the skull (lateral view) suggestive of soft tissue swelling in the left parietal region

A plain radiograph of the skull was suggestive of soft tissue swelling in the left parietal region with bone rarefaction (Figure 1).

His complete blood investigations, liver function tests, renal function tests, and chest radiographs were all within normal limits except for a raised erythrocyte sedimentation rate (63 mm at the end of the first hour) and a positive Mantoux test.

**Figure 2:**
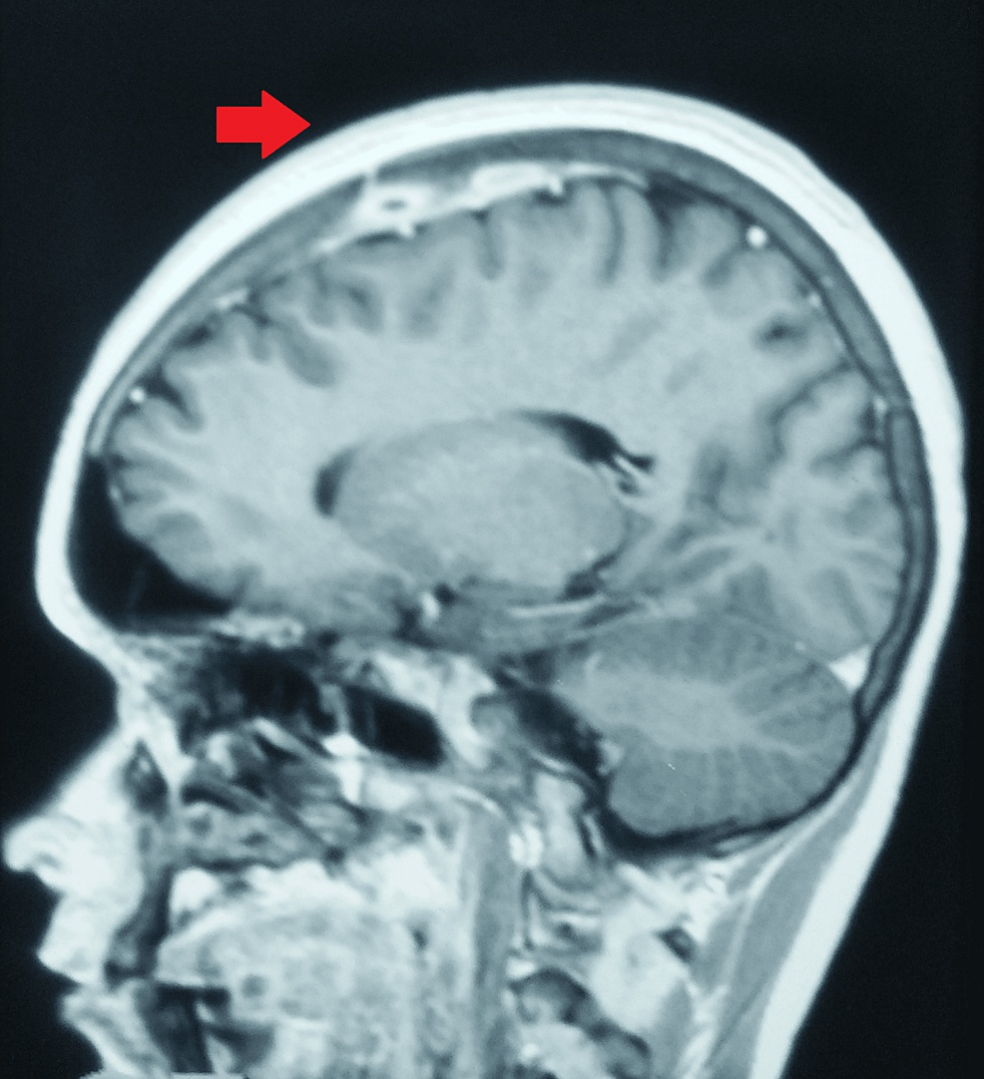
MRI showing a well-defined lesion situated on the left parietal bone MRI: Magnetic resonance imaging

**Figure 3:**
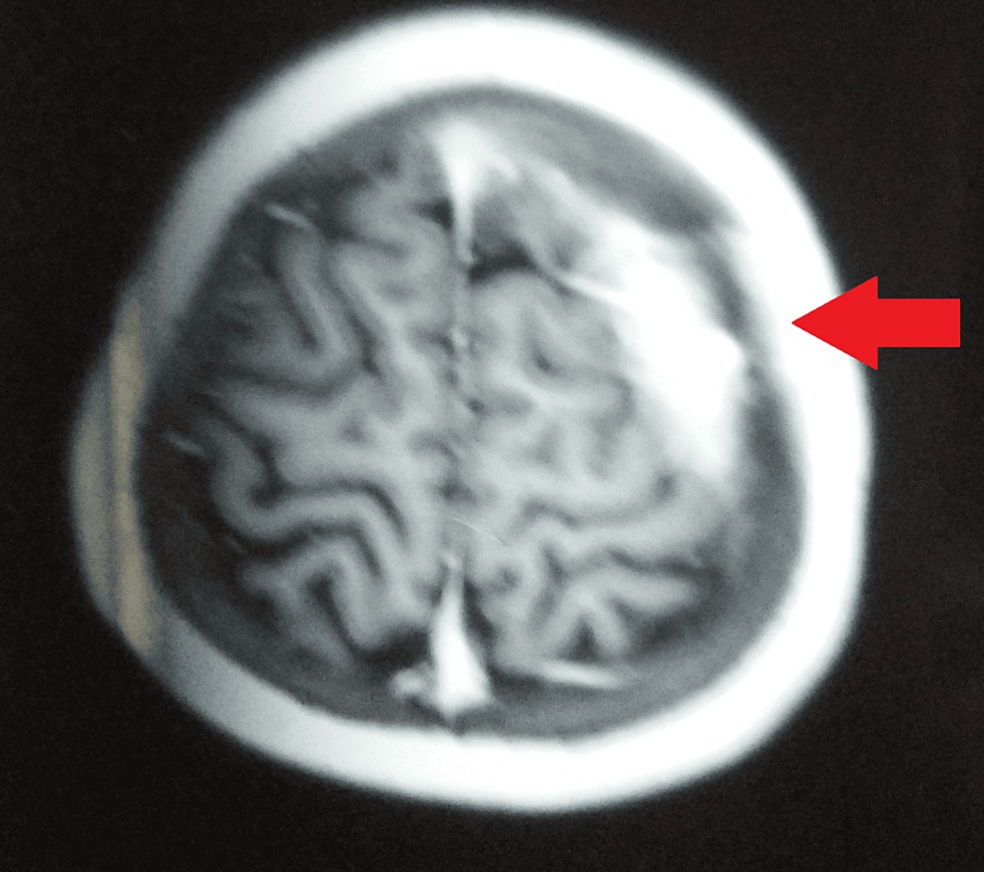
MRI showing the lesion situated on the left parietal bone and extradural abscess MRI: Magnetic resonance imaging

A non-contrast computed tomography of the head revealed a left high parietal, subgaleal, and peripherally enhancing extradural abscess with bony destruction. Magnetic resonance imaging revealed a T2 hyperintense, clear-cut lesion situated on the left parietal bone and an extradural heterointense abscess/lesion under the bony defect. However, there was no evidence of intracranial extension (Figures 2, 3).

An ultrasound-guided biopsy of the parietal bone lesion was performed, and the sample underwent testing for acid-fast bacilli staining, Truenat MTB-RIF assay, and culture. The results indicated necrotizing granulomatous inflammation with a few Langhan’s giant cells suggestive of tuberculosis. The Truenat MTB-RIF assay detected *Mycobacterium tuberculosis* (4.2 x 10^5 ^colony-forming units (CFU)/mL) with no resistance. However, the culture was negative. He received antituberculous treatment, including rifampicin (10 mg/kg), ethambutol (15 mg/kg), isoniazid (5 mg/kg), and pyrazinamide (25 mg/kg), for a duration of two months. This was followed by rifampicin (10 mg/kg), ethambutol (15 mg/kg), and isoniazid (5 mg/kg) for the subsequent 10 months. Over the course of treatment, the swelling decreased, and complete resolution of the scalp lesion was observed at eight months of treatment.

## Discussion

Over the past decade, there has been a significant shift in the clinical patterns and presentations of tuberculosis [3]. Disseminated tuberculosis can arise from either the activation of a latent infection focus or as a consequence of progressive primary pulmonary tuberculosis. It is suggested that tubercular bacilli erode the epithelial layer of the alveoli, entering the pulmonary vein [4]. Subsequently, they travel to the left side of the heart and disseminate into the systemic circulation, ending up in various organs [3].

Reid et al. were the first to describe calvarial tuberculosis in 1842 [5]. It typically arises as a result of primary tubercular infections originating from various sites, such as the lungs, lymph nodes, bones, gastrointestinal tract, or central nervous system. Isolated calvarial tuberculosis, as detailed in the present case, is highly uncommon [3]. The frontal and parietal bones are most commonly affected due to their fairly higher percentage of cancellous bone, followed by the occipital and sphenoid bones [5].

The mode of spread of bacteria is mostly hematogenous, but direct inoculation post-traumatic trauma, as seen in this case, is also a significant cause [2,3]. Diagnosis is difficult mainly due to non-specific presentations on the radiographs, especially in the absence of a history of tuberculosis [3,5]. Besides, establishing the presence of *Mycobacterium tuberculosis* with microbiological and histological confirmation, as done in the present case, is imperative [5]. Nevertheless, advanced radiometric investigations like computed tomography and magnetic resonance imaging can be used for a detailed assessment and diagnosis [3].

Management is essentially medical, with antituberculous chemotherapy four antituberculous drugs for a total of 12 months, and then clinical assessment for a further extension of treatment [2,6]. Currently, surgery is typically reserved for patients presenting with significant factors such as large epidural collections, extensive bone destruction, the formation of a sequestrum, or instances of mass effects on the brain resulting in focal neurological deficits. Surgical intervention involves the removal of sinus tracts along with en-bloc removal of the damaged bone and the excision of granulation tissue [2,3,7].

Isolated reports have indicated instances of calvarial tuberculosis resulting from direct extension from a nearby focus of infection [8].

In a study on 42 cases of calvarial tuberculosis, Raut et al. concluded that individuals with an age range of 11 to 20 years old accounted for the largest proportion of infected patients, or 61.2% [9]. Similarly, Diyora et al. reported a high number of cases in the age group less than 20 years [10]. A case similar to the present case was reported by Nair et al. (2015) [5]. The present case shares similarities with theirs in the absence of constitutional signs of tuberculosis, the presence of a discharging sinus, no neurological deficits, diagnosis by biopsy and histopathology of the lesion of the bone, raised erythrocyte sedimentation rates, and a positive Mantoux test. However, the present case differs from theirs in age, location of the lesion, absence of lesions in the lungs, and conservative management with the antituberculosis drugs.

## Conclusions

A rare case of parietal bone tuberculosis in an Indian boy was presented. The case emphasizes the importance of timely diagnosis of such rare presentations, even in endemic countries. This case also stresses the need for the dissemination of knowledge about such a difficult diagnosis where radiological presentations could be non-specific and the condition mimics other diseases of the skull. It is also important to timely initiate such cases with medical management, as delays could prove fatal.

## References

[1] Wang X, Zhang Q, Wu W (2020). Unusual tuberculosis of the frontal bone: a case report. Open J Intern Med.

[2] Kommu VR, Khera R, Tagore R, Murthy SS, Sundaram C, Kotha S (2019). Tuberculous osteomyelitis of frontal bone: a case study and review of the literature. J Case Rep.

[3] Nazneen J, Patil V, Agarwal U, Karbhari A, Bornak G, Rai P, Mahajan A (2023). Tuberculosis of frontal bone—a rare entity: case report and review of literature. Indian J Med Paediatr Oncol.

[4] Khan FY (2019). Review of literature on disseminated tuberculosis with emphasis on the focused diagnostic workup. J Family Community Med.

[5] Nair AP, Mehrotra A, Das KK, Kumar B, Srivastav AK, Sahu RN, Kumar R (2015). Calvarial tuberculosis of the parietal bone: a rare complication after dental extraction. Asian J Neurosurg.

[6] (2024). Training module on extrapulmonary tuberculosis: standard treatment workflow. https://tbcindia.gov.in/WriteReadData/l892s/7702334778Training_Module_on_Extrapulmonary_TB_-_Book_24032023.pdf.

[7] Shahat AH, Rahman NU, Obaideen AM, Ahmed I, Zahman Au (2004). Cranial-epidural tuberculosis presenting as a scalp swelling. Surg Neurol.

[8] Barton CJ (1961). Tubercolosis of the vault of the skull. Br J Radiol.

[9] Raut AA, Nagar AM, Muzumdar D, Chawla AJ, Narlawar RS, Fattepurkar S, Bhatgadde VL (2004). Imaging features of calvarial tuberculosis: a study of 42 cases. AJNR Am J Neuroradiol.

[10] Diyora B, Kumar R, Modgi R, Sharma A (2009). Calvarial tuberculosis: a report of eleven patients. Neurol India.

